# Combining transcranial electrical stimulation with training in older adults: Effects on dual-task ability

**DOI:** 10.1016/j.neurot.2026.e00889

**Published:** 2026-03-20

**Authors:** Yong Jiang, Azadeh Lesani, Xue Guo, Isabella Wistuba, Raquel Guiomar, Denis Glage, Perianen Ramasawmy, Andrea Antal

**Affiliations:** a Non-Invasive Brain Stimulation Lab, Department of Neurology, University Medical Center Göttingen, Georg-August University of Göttingen, Göttingen, Germany; b Faculty of Physical Education and Sport Sciences, University of Tehran, Tehran, Iran; c University of Coimbra (Portugal), Center for Research in Neuropsychology and Cognitive and Behavioral Intervention (CINEICC), Faculty of Psychology and Educational Sciences, Coimbra, Portugal; d Institute for Sports Sciences, Georg-August University of Göttingen, Göttingen, Germany

**Keywords:** Multitasking, Non-invasive brain stimulation, Healthy elderly, Task training

## Abstract

**Background:**

Enhancing dual-task (DT) performance in older adults through cognitive improvement may help prevent neurodegenerative decline.

**Objective:**

This study aimed to investigate the efficacy of combining repeated transcranial electrical stimulation (tES) targeting the left dorsolateral prefrontal cortex (L-DLPFC) with task training on DT ability in healthy older adults.

**Methods:**

Fifty-eight participants were randomized to receive ten 20-min sessions of cognitive and motor task training concurrently applied with either anodal transcranial direct current stimulation (tDCS), 4 Hz transcranial alternating current stimulation (tACS), or sham tES.

**Results:**

All groups demonstrated significant cognitive improvements under both single- and DT conditions immediately and four weeks after the intervention, with no between-group differences. Although motor dual-task costs decreased across all groups, this did not translate into measurable performance gains.

**Conclusion:**

Overall, tES did not provide significant additive effects on DT performance when combined with multi-session training interventions.

## Introduction

Dual tasking (DT), referring to the ability to perform two tasks simultaneously, represents an essential aspect of human and cognitive functioning. Depending on the nature of component tasks, DT can be categorized into 1) motor-motor, 2) cognitive-cognitive, and 3) cognitive-motor DT, with the latter being most commonly applied in research [1,2]. Impairments in DT performance are generally attributed to competition for limited attentional resources, interference with neural pathways during information processing, or a reduction in overall cognitive capacity [3,4]. The deterioration of DT in old age has been attributed to reduced attention resources and weakened executive function. Such deficits may serve as an early clinical marker of cognitive decline and have been associated with an increased risk of neurodegenerative conditions, particularly Parkinson's disease (PD) [[5], [6], [7], [8], [9], [10]]. Previous studies have suggested that DT-specific decline was considered to be a distinctive functional impairment within the central executive system among the Alzheimer's disease patients [11,12]. Neuroimaging evidence further indicates that higher DT performance is associated with greater neural processing efficiency [13,14], and the close relationship between DT capacity and executive control and attention functions has supported its widespread use in clinical assessment [10,15,16]. Therefore, developing strategies to boost DT in the healthy elderly is becoming increasingly significant, as such interventions holds potential for enhancing well-being and functional independence in older adults. Structured training programs, consisting of targeted motor and cognitive exercises, have been shown to improve DT in the healthy elderly through optimizing motor and/or cognitive function, as well as enhancing coordination/automatization within DT executions [2,17,18]. Low-intensity transcranial electrical stimulation (tES), including transcranial direct current stimulation (tDCS), which modulates cortical excitability by altering neuronal membrane potentials [19], and transcranial alternating current stimulation (tACS), which influences neural synchrony through oscillatory entrainment [20], has been studied for its potential to improve DT performance [[21], [22], [23], [24], [25], [26]]. Studies involving these types of stimulation typically target the left dorsolateral prefrontal cortex (L-DLPFC), given its critical role in cognitive control processes [27,28], such as executive function, which is generally believed to be a fundamental component of performing the DT paradigm [29]. While additional evidence is needed to further verify the effects of a single tDCS session on dual-task ability in older adults, previous studies have reported promising outcomes [17]. While both DT and ST were observed to improve after long-period, multi-session training, DT performance was reported to have improved after a single 20-min tES session [17]. This has led to increasing interest in combining tES with task training and extending stimulation sessions to maximize potential benefits, yet current evidence remains inconclusive. Schneider et al. [30] reported reduced dual task cost (DTC) after a single 20min session of high-definition tDCS at 3 mA over the left primary motor cortex (M1) and L-DLPFC combined with treadmill walking training in the healthy elderly when compared to the sham setting, suggesting a direct stimulation effect. In contrast, Schabrun et al. [31] highlighted the central role of training, as both 9-session 2 mA 20min anodal tDCS over left M1 and sham stimulation combined with repeated 9-session 60min motor-cognitive DT training led to comparable improvements in older PD patients. By comparison, Liao et al. [32] emphasized the role of anodal tDCS over the L-DLPFC, as only the combination of 40min Tai Chi training with 2 mA, 20min tDCS for 36 sessions produced significant improvements in DT performance in mild cognitive impairment patients compared to sham. The most recent study suggested that 2 mA, 20min L-DLPFC tDCS followed by 30min treadmill training for 12 sessions demonstrated greater improvements on cognitive-motor DT, but not on motor-motor DT compared to the sham condition in PD patients [33]. Hence, the effects of combined methods remain to be explored, while the impact of combined tACS and training on DT in the healthy elderly has not been reported.

In this study, we concurrently applied 10 20min sessions of anodal tDCS or 4Hz tACS [34] targeting the L-DLPFC with task training. Our main aim was to investigate the effects of combining tES with task training on DT performance in healthy older adults. Considering the distinct modulatory characteristics of tDCS and tACS, we also evaluated the individual component tasks to examine how changes in DT ability relate to performance under single task (ST) conditions, aiming to further clarify this effect.

## Materials and Methods

The study was approved by the local ethics committee (ID: 4/7/22), according to the Declaration of Helsinki, and was registered at the German Clinical Trials Register under the identification number DRKS00031042. All participants gave verbal and written consent.

### Participants

We recruited participants through local newspapers and flyers. Healthy older adults aged 60–85 years, with a Montreal Cognitive Assessment (MoCA) score of 26 or higher [35], were included in the study. Exclusion criteria included 1) any acute medical condition requiring hospitalization within the past three months; 2) metal implants in head or pacemakers, ferromagnetic objects, or other objects with risk of contact with the electrodes; 3) use of medications affecting the central nervous system; 4) acute cardiovascular disease such as uncontrolled or untreated high or low blood pressure; 5) presence of lower-extremity pain, musculoskeletal disorders, or any other condition that may influence gait and balance; 6) diagnosis of any major neuropsychiatric disorders including drug/alcohol addiction, 7) visual impairments, e.g., glaucoma.

Sample size calculation using the G∗Power 3.1 software (F tests, ANOVA: Repeated measures, within-between interaction) with the following parameters: a small effect size of 0.25, an α error rate of 0.05, a statistical power of 0.95, and a correlation among repeated measures of 0.50. According to the calculation, a minimum of 18 participants per group was required.

### Study design

This randomized, sham-controlled, double-blinded study was conducted at the University Medical Center Göttingen, Germany, from June 2023 to July 2025. Participants were randomly allocated to receive 10 20-min sessions (within four weeks) of task training concurrently applied with either 1) anodal tDCS of the L-DLPFC at 2 mA (tDCS + T), 2) 4 Hz tACS targeting the L-DLPFC at 2 mA (tACS + T), or 3) sham tES (Sham + T). The sham intervention was categorized into sham tACS and sham tDCS. To achieve this, six stimulators were coded in the study. Two stimulators were programmed for each stimulation condition under the same code but with different numbers (e.g., A1, A2 for anodal tDCS, B1, B2 for sham tDCS, and sham tACS). A block randomization with stratification by sex, age, height, and body weight was conducted by an independent researcher who was not involved in data collection or analysis. The colleague pre-programmed the stimulators according to the protocol. Both the investigators and the participants were blinded to the stimulation type. Data analysis was also conducted in a blinded manner.

### Transcranial electrical stimulation

tES was applied for 20 min in 10 sessions delivered within four weeks (minimum three sessions per week) using a CE-certified battery-driven stimulator (NeuroConn GmbH, Ilmenau, Germany). A pair of 5 cm × 5 cm conductive rubber electrodes was placed on the participant's scalp using conductive cream (AC cream, GVB geliMed GmbH, Germany). Electrode placement followed the F3-FP2 montage using the international 10–20 EEG system. For anodal tDCS of the L-DLPFC, a constant current of 2 mA was applied for 20 min. Active tACS was delivered at 4 Hz at a peak-to-peak amplitude of 2 mA for 20 min. For both tDCS and tACS, there was a 15s ramp-up and 15s ramp-down. Sham conditions were programmed for a 30s stimulation period with a 15s fade-in/out duration.

To assess the safety of the combined intervention, participants were asked to report the occurrence of any side effects (e.g., tingling, skin redness) and adverse events (e.g., headache, dizziness, fatigue, nervousness, sleep problems) before and after each intervention session. At PA, participants were asked to guess whether they received active or sham stimulation. They were informed of the allocated group after all follow-ups were completed.

### Task training program

Each stimulation session was accompanied by a structured training program. The program consisted of five 20 min cognitive training sessions based on the N-back paradigm, followed by five 20 min low-intensity motor training sessions. The cognitive training served as the core component to strengthen working memory, divided attention, and response coordination. The motor training was incorporated to enhance postural control and motor execution, while simultaneously minimizing direct task overlap and near transfer with the DT assessment.

**Fig. 1:**
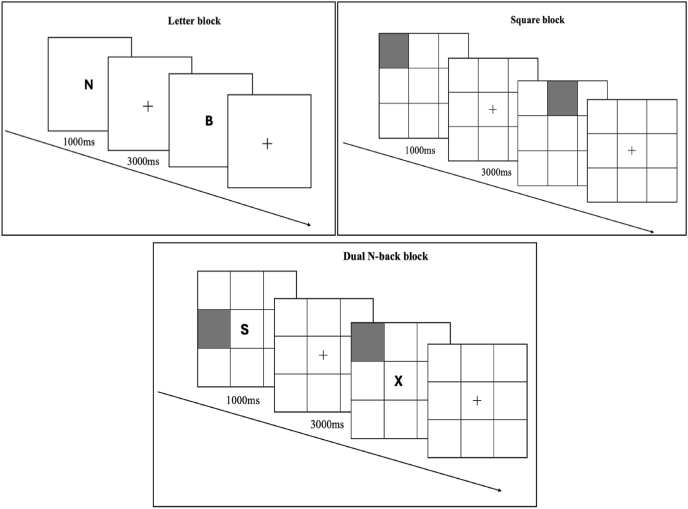
The illustration of three different blocks within the N-back training program. During letter blocks, participants responded with left-Shift only. In square blocks, only right-Shift was applied. In dual N-back blocks, left-Shift and right-Shift were recruited.

During the cognitive training sessions, participants were seated in a comfortable position and instructed to perform N-back task on a 24-inch monitor (refer to Fig. 1 for an overview of task). The training was conducted via PEBL2. The difficulty was gradually increased from 1-back to 2-back. To further challenge and train divided attention and executive control, a dual-N-back block was included in every cognitive training session. More specifically, we applied letters and squares in different blocks during each session. Participants responded with left-Shift in letter blocks under 1-back/2-back conditions, where the letter was present for 1000 ms, with the interstimulus interval ranging from 3000 ms to 5000 ms according to individuals’ abilities. In square blocks, a nine-square grid was presented on the screen center, and a square randomly appeared in one of the eight peripheral positions. Participants responded with right-Shift in the 1-back and 2-back conditions. In dual-N-back blocks, squares and letters were presented simultaneously, while letters always appeared in the center of the nine-square grid. The left-Shift was assigned to letters, and the right-Shift was reserved for squares. In 1-back conditions, for instance, only the left-Shift should be pressed when the letters were the same as the previous one, while the position of the square was different. Conversely, the right shift should be pressed individually in the opposite scenario. Both buttons should be pressed when both conditions were met.

The low-intensity motor training program consisted of three parts in each session: a 4-min warm-up, 12 min of balance and standing training, and a 4-min relaxation period. The stimulator was fixed in the waist of the participants. Specifically, a cognitively demanding motor task was employed to engage higher-level motor control, as detailed in Table 1.

**Table 1:** Overview of motor training program.

Phase	Duration	Content	Specific Exercises
Warm-up	4 min	Whole-body stretching and joint mobilization	- Whole-body stretching - Ankle warm-up - Knee warm-up
Balance and standing training	12 min	Balance exercises performed barefoot or using a Bosu ball	**Single-/double-leg standing**: Standing on the ground and the Bosu ball with one/two legs **Stair-climbing**: • Start from the ground with the ball in front. • Step up onto the ball in a stair-like manner. • Step backward off the ball. **Up and over**: • Start from standing on the ground with the ball to the left or right. • Step laterally onto the ball, continue stepping off in the same direction. • Return to the starting position. Support was offered if needed. **Standing scale (dynamic balance):** • Stand on the Bosu ball with one foot while holding a handrail with the opposite hand for support. • Lift the free leg forward, bending the knee, and bring it toward the opposite elbow in front of the torso. • Lean the trunk forward while extending the lifted leg backward and the opposite arm forward simultaneously, forming a straight line from the fingertips to the heel (viewed from the side, the body resembles the letter “T”). • Maintain balance briefly, then return to the upright standing position and repeat as required. **Cognitive-demanding stepping**: Condition 1: • Stand on the ground facing the Bosu ball and march in place. • Step onto the ball with the left or right foot according to the verbal instruction (German). (“Links Hoch” = left foot up; “Rechts Hoch” = right foot up) • Place the other foot onto the ball and continue marching in place while balancing on the ball. • Step backward down from the ball with the instructed foot. (“Links Unten” = left foot down; “Rechts Unten” = right foot down) • Return both feet to the ground and resume marching in place. Condition 2: • Participants respond with the limb opposite to the given instruction (e.g., “Links Hoch” = step up with the right foot; “Rechts Unten” = step down with the left foot). • Marching in place was maintained throughout. • Rest breaks were taken as required.
Relaxing	4 min	Stretching and cool-down	- Stretching and relaxation exercises

### Outcome measures

Measurement visits took place at baseline (BA), within 24 h following the last intervention (PA), and four weeks following the last intervention (FU). The primary parameters were the DT measurements, which consisted of 1) concurrent 20-m walking combined with serial subtraction by sevens from a three-digit number (walking-subtraction), 2) concurrent eyes-open standing and subtraction task (EO-standing-subtraction), and 3) concurrent eyes-closed standing and subtraction task (EC-standing-subtraction). Previous research has demonstrated that the presence of visual inputs positively impacts balance maintenance, whereas their absence facilitates attention and aids in focusing on non-visual external tasks or internal thoughts [36]. Hence, eyes-open (EO) and eyes-closed (EC) conditions were applied for standing-subtraction tasks. EO-standing-subtraction refers to DT with low motor load but high cognitive load, whereas EC-standing-subtraction refers to DT with high motor load but low cognitive load. The secondary outcomes included DTC and the performance of component tasks within the ST condition, including walking speed, calculation speed and accuracy, and sway distances during standing. At each assessment visit, participants completed both DT and ST conditions as part of a standardized assessment battery.

### Primary outcomes

The walking-subtraction DT was performed in a 10-m marked corridor. Participants walked at their natural, self-preferred pace, turned around at the end, and returned. The same neutral instruction (“please walk at your normal, comfortable pace”) was given at every assessment (BA, PA, FU) to maintain consistency across sessions. Meanwhile, they performed the subtraction task while walking back and forth until completing ten consecutive subtractions. The time required to complete the walk and the performance on the subtraction task were documented separately. For the subtraction task, participants were given a random three-digit number (299–999) and asked to perform 10 consecutive subtractions of seven. For standing-subtraction DTs, the time required to complete the subtraction task under EO and EC conditions and the sway distance of the center of pressure were recorded. Standing-related tasks were assessed using a three-dimensional force plate (9260AA6, 1500Hz, Kistler, U.S.A.) in conjunction with MYOMuscle software (Noraxon, version 3.6, U.S.A.). Participants were asked to stand still on the force plate with feet parallel and pointing forward, arms relaxed at the sides, and eyes either open and directed forward or closed, depending on the condition. The time to complete subtraction tasks was documented, as well as the accuracy, during which the sway distances of the center of pressure were measured. In sum, the primary outcomes were: 1) walking and subtraction performance under walking-subtraction, 2) sway distance of the center of pressure and subtraction performance within EO-standing-subtraction, and 3) sway distance of the center of pressure and subtraction performance in EC-standing-subtraction DTs. In addition, surface electromyography (MyoTrace 400, Noraxon, U.S.A., Inc., synchronized with the Kistler system) was applied to the dominant leg's tibialis anterior and soleus muscles to capture muscle activity. The recording unit was secured at the participant's waist.

### Secondary outcomes

DTC refers to the percentage change in the parameters between DT and ST. Here, we applied the same formula as described by Manor [22].

The performance of component tasks within ST conditions: The 20-m walking task was conducted in the same corridor like before, following the same requirements and identical instruction which employed in DT walking. The subtraction task was conducted while seated. EO and EC standing tasks were conducted with the corresponding postural requirements. Each standing trial lasted 1min, and sway distances were recorded with the synchronized electromyography.

### Self-report questionnaires

The German version of the Falls Efficacy Scale–International (FES-I) [37] was used to evaluate participants’ concern about falling during a range of daily activities.

The 36-Item Short Form Health Survey (SF-36, Cronbach's alpha = 0.87) [38] was used to assess overall health-related quality of life across eight domains: physical functioning, role limitations due to physical health, bodily pain, general health, vitality, social functioning, role limitations due to emotional problems, and mental health.

### Statistical analyses

A one-way ANOVA was performed on demographic variables to confirm group comparability. Homogeneity of variance was checked using Levene's test. For each outcome variable, the distribution within every Group∗Time combination was examined with the Shapiro–Wilk test. Depending on distributional properties, either linear mixed-effects models (LMMs) or generalized linear mixed-effects models (GLMMs) were applied. The general model specification was: Outcome ∼ Group × Time + Age + Sex + MoCA + (1 | Subject), where Group (tACS + T, tDCS + T, Sham + T) and Time (BA, PA, FU) served as fixed effects, and subject-specific intercepts were modeled as random effects. This model was designed to primarily evaluate intervention-related effects, with a particular focus on the Group × Time interaction. Age, sex, and MoCA were included as covariates a priori to control for their known influences on cognitive and motor performance in older adults. These variables were treated as nuisance covariates and were included consistently across all models. Random slopes for time were initially included but were removed if convergence problems or singular fits occurred; in such cases, a simpler random-intercept model or, if necessary, a fixed-effects model was fitted instead.

For missing data, outcomes were analyzed both without imputation and with several imputation methods, including mean, median, random forest, and predictive mean matching. Overall missingness was low, accounting for approximately 2.0% of all outcome data points (125 out of 6270 observations), with no systematic pattern across groups or assessment time points. Among these approaches, the analysis based on the original dataset—containing missing values—produced the best-fitting model, as indicated by the lowest Akaike Information Criterion (AIC) [39]. Therefore, all the statistical analyses were performed with original data. Post hoc analysis with Bonferroni correction was performed when significant effects or interactions were found. The effect size was reported as the partial eta squared η^2^ (ηp^2^) with LMM [40], while the marginal and conditional R^2^ (R^2^m, R^2^c) were reported for those with GLMM. Furthermore, baseline values were included as covariates for parameters showing either marginal or significant differences at baseline (Outcome ∼ Group × Time + Age + Sex + MoCA + Baseline, with random intercept).

To assess the blinding effect, we employed the Chi-squared (χ^2^) test to ascertain whether the participants’ estimations of the type of stimulation they received deviated from the anticipated probability (50%).

Descriptive statistics, one-way ANOVA for demographic comparisons, Levene's test for homogeneity of variance, Shapiro–Wilk tests for normality assessment, and Chi-squared tests for the evaluation of blinding efficacy were performed using IBM SPSS Statistics 20.0. LMM and GLMM were conducted using R (version 4.5.1) and RStudio with packages *lme4* and *glmmTMB* (for more details please refer to Supplementary Table 3).

## Results

### Participants

**Fig. 2:**
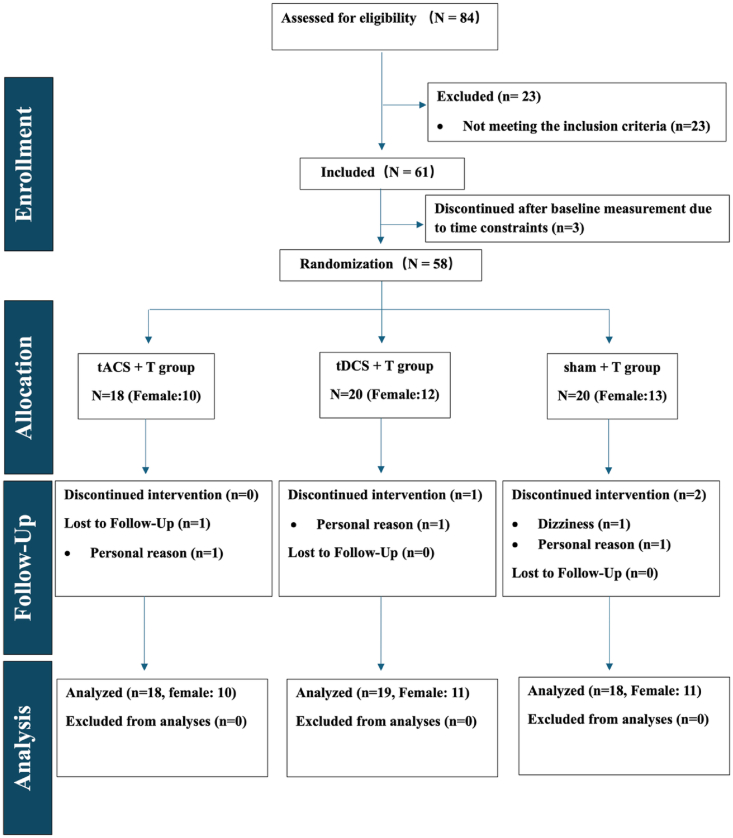
Consolidated Standards of Reporting Trials flow diagram of participants in the study, comparing concurrent tACS combined with training group (tACS + T), concurrent tDCS combined with training group (tDCS + T), and concurrent sham tES combined with training (Sham + T) groups.

Out of 84 screened interested participants, 61 (72.61%) met the inclusion criteria. Three participants left the study after the baseline assessment due to time constraints (Fig. 2). Thus, 58 participants were randomly allocated to the three intervention groups: ten 20min sessions of 1) 4 Hz tACS combined with task training (tACS + T), 2) anodal L-DLPFC tDCS with task training (tDCS + T), and 3) sham tES paired with task training (sham + T). Three participants discontinued the intervention due to either personal reasons or experienced side effects of stimulation. Fifty-five participants, who completed the intervention, were included in the analysis. Only one participant in the tACS + T group dropped out during follow-up due to personal reasons. There were no significant differences in baseline demographics among the three groups (Table 2).

**Table 2:** Demographics of participants in three groups at baseline.

Demographics	Sham + T	tACS + T	tDCS + T	*P*-value
Age, years	71.06 ± 5.03	72.26 ± 5.71	71.94 ± 5.51	0.48
Sex (F/M)	10/8	11/8	11/7	–
Height (cm)	171.39 ± 8.74	169.79 ± 9.31	168.94 ± 8.41	0.35
Body weight (kg)	74.94 ± 11.83	73.21 ± 14.88	71.50 ± 10.71	0.38
MoCA	26.67 ± 1.25	28.05 ± 1.50	27.89 ± 1.40	0.71
Exercise hour (h/week)	3.66 ± 3.17	3.61 ± 1.98	3.63 ± 1.97	0.99

Data are presented as mean and standard deviation (SD), unless otherwise specified. MoCA: Montreal Cognitive Assessment test.

### Primary outcomes

#### Walking-subtraction dual task

**Fig. 3:**
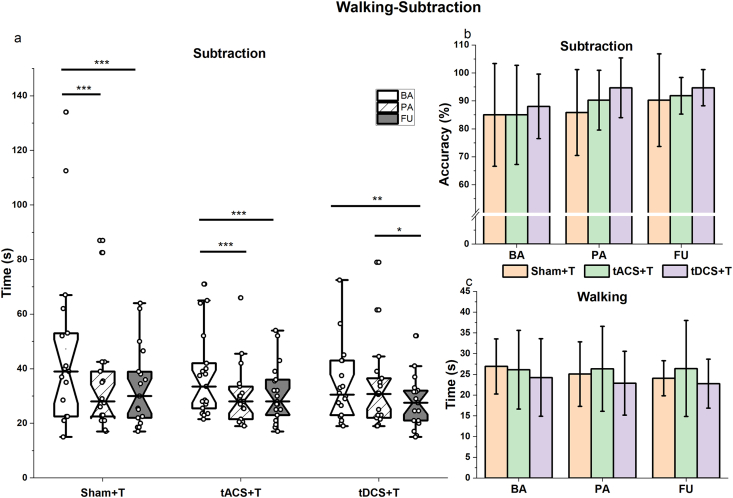
Walking and subtraction performances under the walking-subtraction condition. Box plots with different internal fill patterns represent various assessment points, while colored columns denote different groups. **a**: The time to complete the subtraction task while performing the walking task. Notched box plots show the median, interquartile range, and 95% confidence interval of the median; dots represent individual data points. **b**: The accuracy of the subtraction task. **c**: Time to complete 20 m walking while performing the subtraction task. In panels b and c, bars indicate mean values and error bars represent ±standard deviation ∗: 0.01< p ≤ 0.05; ∗∗: 0.001 < p ≤ 0.01; ∗∗∗: p ≤ 0.001.

We found a significant Group∗Time interaction (χ^2^ (4) = 9.59, p = 0.04, R^2^m = 0.76, R^2^c = 0.81) for the time to complete the subtraction task under the walking-subtraction condition. Further investigation into the interaction effects revealed simple Time effects, with faster calculation speeds being demonstrated in all groups (Fig. 3a). Both the sham + T and tACS + T groups exhibited higher calculation speed after the intervention (PA) (sham + T: estimate = −0.25, SE = 0.06, p < 0.001; tACS + T: estimate = −0.18, SE = 0.05, p = 0.005) and FU (Sham + T: estimate = −0.28, SE = 0.06, p < 0.001; tACS + T: estimate = −0.16, SE = 0.05, p = 0.012), compared to the baseline (BA). However, for the tDCS + T group, the calculation speed was only significantly lower at the four-week following the last combined intervention (FU) compared to BA (estimate = −0.20, SE = 0.06, p = 0.002), and PA (estimate = −0.17, SE = 0.06, p = 0.017). No group differences were observed for the improvement in calculation speed.

No significant interaction or main effect of time was noted for the subtraction accuracy or walking speed (Fig. 3b and c, Table 3).

**Table 3:** Results of primary outcomes under walking-subtraction, EO-standing-subtraction, and EC-standing-subtraction conditions.

Parameters	Baseline control^a^	Sham + T			tACS + T			tDCS + T			Interaction	Group effect	Time effect	R^2^m	R^2^c
		BA	PA	FU	BA	PA	FU	BA	PA	FU					
DTwalking (s)	N	26.90 ± 6.64	25.06 ± 7.78	24.08 ± 4.23	26.11 ± 9.51	26.33 ± 10.23	26.41 ± 11.55	24.22 ± 9.34	22.87 ± 7.71	22.77 ± 5.91	χ^2^ (4) = 3.04, P = 0.55	χ^2^ (2) = 2.91, p = 0.23	**χ^2^ (2)** = **6.11, p** = **0.047**^b^	0.20	0.80
DTsubT (s)	Y	47.23 ± 32.45	34.41 ± 19.95	33.27 ± 14.46	37.39 ± 15.29	30.33 ± 11.19	30.76 ± 10.50	34.08 ± 13.88	32.98 ± 15.75	27.82 ± 9.76	**χ^2^ (4)** = **9.59, p** = **0.047**	χ^2^ (2) = 0.54, p = 0.76	**χ^2^ (2)** = **25.82, p** < **0.001**	0.76	0.81
DTsubACC	N	0.85 ± 0.18	0.86 ± 0.17	0.90 ± 0.11	0.85 ± 0.15	0.90 ± 0.10	0.91 ± 0.10	0.88 ± 0.16	0.94 ± 0.06	0.94 ± 0.06	χ^2^ (4) = 1.41, P = 0.84	χ^2^ (2) = 0.85, P = 0.65	χ^2^ (2) = 2.69 p = 0.26	0.18	0.27
DT EO-standing (mm)	N	1356.81 ± 405.32	1318.60 ± 439.91	1386.85 ± 388.28	1288.12 ± 395.50	1311.54 ± 391.55	1322.01 ± 321.67	1491.49 ± 430.96	1499.50 ± 365.84	1432.97 ± 325.70	χ^2^ (4) = 5.56, P = 0.23	χ^2^ (2) = 2.33, p = 0.31	χ^2^ (2) = 1.74, p = 0.41	0.06	0.91
Tibialis anterior (uv)	N	12.94 ± 10.86	14.65 ± 11.21	10.71 ± 5.26	10.64 ± 9.51	11.28 ± 9.09	9.50 ± 5.51	11.50 ± 6.06	12.91 ± 10.08	9.99 ± 5.86	χ^2^ (4) = 0.99, P = 0.91	χ^2^ (2) = 3.08, p = 0.21	χ^2^ (2) = 2.14, p = 0.34	0.07	0.53
Soleus (uv)	Y	15.20 ± 10.42	20.91 ± 10.04	20.46 ± 17.18	13.12 ± 10.45	15.02 ± 7.59	15.84 ± 7.58	18.06 ± 9.37	19.26 ± 9.07	16.68 ± 8.85	χ^2^ (4) = 5.03, p = 0.28	χ^2^ (2) = 2.69, p = 0.26	**χ^2^ (2)** = **10.23, p** = **0.006**	0.20	0.56
DT EC-standing (mm)	N	1459.63 ± 355.63	1436.82 ± 374.70	1467.77 ± 468.79	1428.51 ± 331.15	1513.83 ± 381.30	1436.18 ± 344.32	1581.21 ± 360.11	1576.22 ± 390.37	1607.43 ± 364.08	χ^2^ (4) = 4.92, p = 0.29	χ^2^ (2) = 1.79, p = 0.40	χ^2^ (2) = 0.49, p = 0.78	0.08	0.90
Tibialis anterior (uv)	N	13.43 ± 9.38	15.42 ± 15.31	12.91 ± 6.32	13.77 ± 18.43	15.46 ± 21.50	16.36 ± 19.25	14.45 ± 11.28	13.99 ± 9.00	12.59 ± 8.75	χ^2^ (4) = 2.57, p = 0.63	χ^2^ (2) = 2.41, p = 0.29	χ^2^ (2) = 0.05, p = 0.97	0.10	0.60
Soleus (uv)	N	15.20 ± 10.41	21.27 ± 10.45	23.08 ± 17.339	13.24 ± 9.94	14.74 ± 7.23	17.12 ± 8.76	18.38 ± 9.01	19.16 ± 8.54	16.36 ± 8.25	χ^2^ (4) = 6.79, p = 0.14	χ^2^ (2) = 4.71, p = 0.09	**χ^2^ (2)** = **7.14, p** = **0.03**^b^	0.18	0.48
DT EOsubT (s)	Y	45.33 ± 26.19	35.72 ± 18.04	31.72 ± 13.44	37.60 ± 12.39	33.23 ± 11.69	29.76 ± 7.71	31.94 ± 8.19	33.93 ± 16.49	27.32 ± 9.02	**χ^2^ (4)** = **12.27, p** = **0.015**	χ^2^ (2) = 1.26, p = 0.52	**χ^2^ (2)** = **40.96, P** < **0.001**	0.77	0.86
DT EOsubAcc	Y	0.88 ± 0.15	0.94 ± 0.09	0.96 ± 0.06	0.93 ± 0.07	0.93 ± 0.07	0.95 ± 0.07	0.97 ± 0.04	0.97 ± 0.03	0.96 ± 0.04	**χ^2^ (4)** = **2.62, p** = **0.03**	**χ^2^** (2) = 2.30, P = 0.10,	**χ^2^ (2)** = **8.33, P** < **0.001**	0.46	0.50
DT ECsubT (s)	Y	40.25 ± 21.99	35.69 ± 20.84	31.77 ± 12.38	36.73 ± 13.29	30.42 ± 10.73	28.89 ± 13.07	32.33 ± 7.95	31.03 ± 8.87	28.64 ± 9.49	**χ^2^ (4)** = **5.28, p** = **0.26**	χ^2^ (2) = 0.64, p = 0.72	**χ^2^ (2)** = **13.23, p** = **0.001**	0.81	0.86
DT ECsubAcc	N	0.92 ± 0.13	0.97 ± 0.05	0.96 ± 0.07	0.93 ± 0.07	0.94 ± 0.08	0.93 ± 0.09	0.95 ± 0.05	0.95 ± 0.05	0.96 ± 0.04	χ^2^ (4) = 3.24, p = 0.51	χ^2^ (2) = 1.59, p = 0.44	χ^2^ (2) = 4.57, p = 0.10	0.07	0.20

a Baseline data as co-various or not.

b Non-significant in post-hoc; DTsubT: the time to complete subtraction task in walking-subtraction task; DTsubACC: the accuracy of subtraction task under walking-subtraction task; DT EO-standing: displacement of the center of pressure under the standing-subtraction task with eyes-opened (EO); DT EC-standing: displacement of the center of pressure under the standing-subtraction task with eyes-closed (EC); DT EOsubT: the time to complete subtraction task in EO-standing-subtraction task; DT EOsubAcc: the accuracy of subtraction task under EO-standing-subtraction task; DT ECsubT: the time to complete subtraction task in EC-standing-subtraction task; DT ECsubAcc: the accuracy of subtraction task under EC-standing-subtraction task.

#### Eyes-opened (EO)-standing-subtraction dual task

**Fig. 4:**
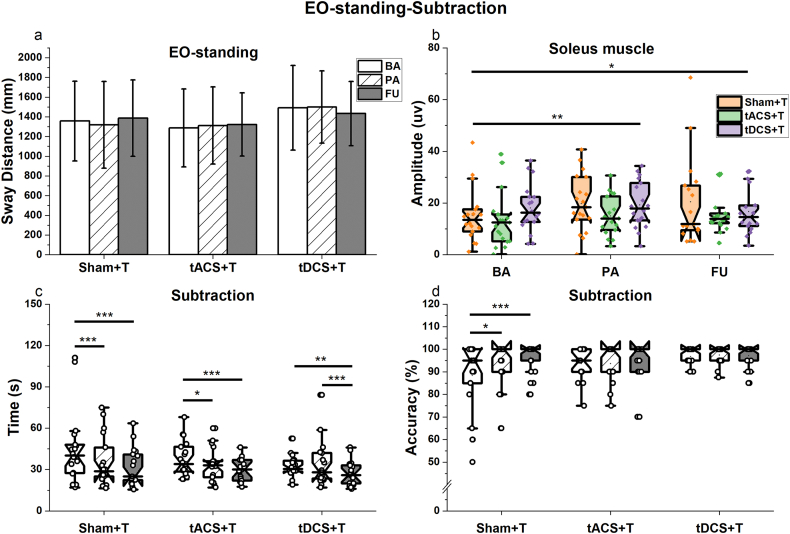
Balance and subtraction performances within the EO-standing-subtraction task. Black and white columns or box plots with different internal fill patterns represent various assessment points, while colored box plots denote different groups. **a**: Sway distance of the center of pressure. No intervention effect was observed regarding the sway distance during EO-standing while performing a subtraction task. Error bars represent mean ± standard deviation. **b**: Mean amplitude of the Soleus muscle. Higher mean amplitude of the soleus muscle during EO-standing DT was observed after intervention and throughout FU. **c**: The time to complete subtraction task while performing EO-standing. An interaction (p = 0.02) was observed, increased performance was demonstrated immediately after the intervention in the sham + T and tACS + T groups, while improvement in the tDCS + T group was observed at FU. **d**: The accuracy of the subtraction task. An interaction (p = 0.03) was observed, the sham + T group demonstrated better performance after the intervention. In panels b, c, and d, notched box plots show the median, interquartile range, and 95% confidence interval of the median; dots represent individual data points. ∗: 0.01< p ≤ 0.05; ∗∗: 0.001 < p ≤ 0.01; ∗∗∗: p ≤ 0.001.

No intervention effects on standing performance were observed under the EO-standing DT condition (Fig. 4a). However, we found a main effect of time (χ^2^ (2) = 10.23, p = 0.006, R^2^m = 0.20, R^2^c = 0.56) for the activation of the soleus muscle of the dominant leg (Fig. 4b). All three groups demonstrated increased muscle activation over time, with significant differences between BA and PA (estimate = 0.30, SE = 0.08, p = 0.001), BA and FU (estimate = 0.22, SE = 0.09, p = 0.03). No significant Group∗Time interaction or main effect was noted for activation of the tibialis anterior (Table 3).

A significant Group∗Time interaction was observed on calculation time (χ^2^ (4) = 12.27, p = 0.015, R^2^m = 0.77, R^2^c = 0.86; Fig. 4c). Further analyses revealed faster calculation speeds from BA to PA (Sham + T: estimate = −0.21, SE = 0.05, p < 0.001; tACS + T: estimate = −0.13, SE = 0.05, p = 0.014) and FU (Sham + T: estimate = −0.30, SE = 0.05, p < 0.001; tACS + T: estimate = −0.21, SE = 0.05, p < 0.001) in both the sham + T and tACS + T groups. In the tDCS + T group, a significant reduction of calculation time was identified at FU only (BA to FU: estimate = −0.17, SE = 0.05, p = 0.002; PA to FU: estimate = −0.18, SE = 0.05, p < 0.001). We did not find any significant group differences.

Similarly, an interaction effect on calculation accuracy was found (χ^2^ (4) = 2.62, p = 0.03, R^2^m = 0.46, R^2^c = 0.50). Interestingly, despite the lack of group differences, simple Time effect analyses revealed improved accuracies post-intervention only in the sham + T group (BA to PA: estimate = 0.06, SE = 0.02, p = 0.014; BA to FU: estimate = 0.1, SE = 0.02, p < 0.001), but not in the others (Fig. 4d).

#### Eyes-closed (EC)-standing-subtraction task

**Fig. 5:**
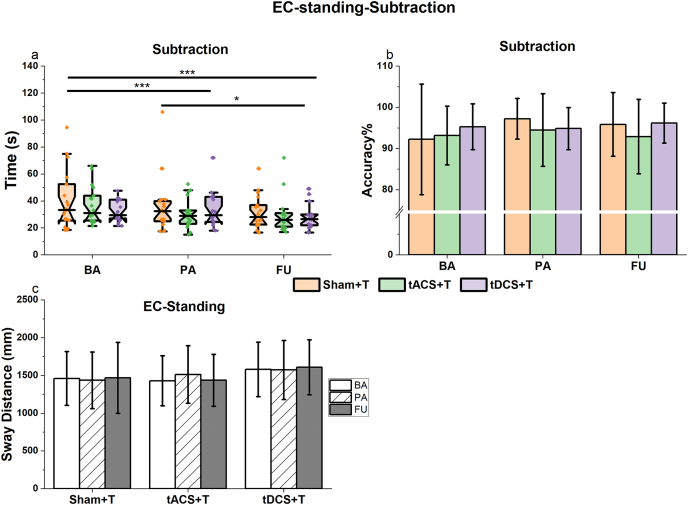
The standing and subtraction performances within the EC-standing-subtraction task. Colored box plots or columns represent various groups, black and white columns denote different assessment points. **a**: Time to complete the subtraction task while performing the EC-standing. Notched box plots show the median, interquartile range, and 95% confidence interval of the median; dots represent individual data points. **b**: The accuracy of the subtraction task. **c**: Sway distance during EC-standing, while performing the subtraction. In panels b and c, error bars represent mean ± standard deviation. ∗: 0.01< p ≤ 0.05; ∗∗∗: p ≤ 0.001.

Although the absence of interaction effect was observed in the subtraction task, the main Time effect (χ^2^ (2) = 13.23, p = 0.001, R^2^m = 0.81, R^2^c = 0.86) suggested an increase in calculation speed over time, independent of group (BA to PA: estimate = -0.11, SE = 0.02, p < 0.001; BA to FU: estimate = -0.18, SE = 0.02, p < 0.001); PA to FU: estimate = -0.07, SE = 0.02, p = 0.03) (Fig. 5a). No other significant effects were noted for subtraction accuracy or sway distance (Fig. 5b and c, Table 3).

Our results supported our task application that EO condition referred to higher cognitive loading (subtraction: EO vs. EC: estimate = -0.03, SE = 0.01, p = 0.04), while EC had higher motor loading (standing: EO vs. EC: estimate = 0.09, SE = 0.01, p < 0.001).

### Secondary outcomes

#### Dual task cost (DTC)

**Fig. 6:**
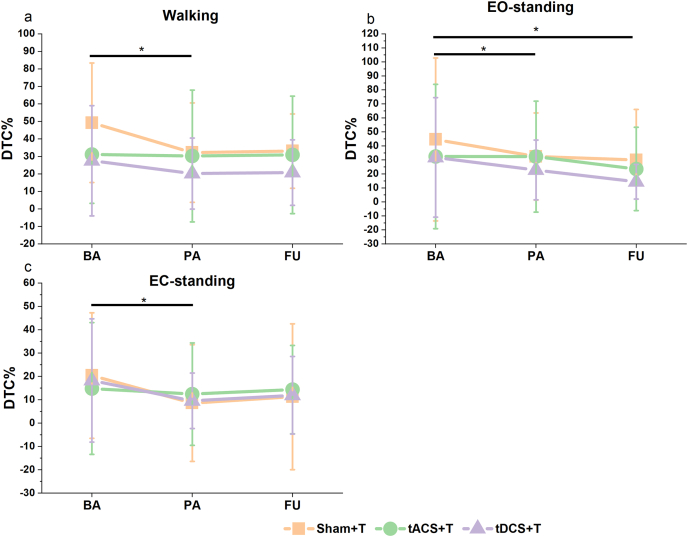
DTCs to motor tasks under DT conditions. Colored lines represent different groups. Error bar represents mean ± standard deviation. All groups demonstrated lower DTC to **a**) walking, **b**) EC-standing, and **c**) EO-standing while performing the subtraction task after intervention, without significant group differences. ∗: 0.01< p ≤ 0.05; ∗∗: 0.001 < p ≤ 0.01; ∗∗∗: p ≤ 0.001.

There was a significant main effect of Time on the DTC to walking (χ^2^ (2) = 10.49, p = 0.005, R^2^m = 0.58, R^2^c = 0.63; Fig. 6a), and DTC to EC-standing (χ^2^ (2) = 6.78, p = 0.03, R^2^m = 0.04, R^2^c = 0.60; Fig. 6b), without any group differences. Post-hoc pairwise comparisons revealed that DTC decreased significantly from BA to PA (DTC_walking_: estimate = −0.08, SE = 0.03, p = 0.047; DTC_EC-standing_: estimate = −0.07, SE = 0.03, p = 0.032). A main effect was also observed on DTC of EO-standing (χ^2^ (2) = 9.56, p = 0.008, R^2^m = 0.03, R^2^c = 0.85; Fig. 6c), pairwise comparisons demonstrated lower DTCs from BA to PA (estimate = - 0.08, SE = 0.03, p = 0.026), and BA to FU (estimate = −0.08, SE = 0.03, p = 0.029). No other significant differences in the DTC of subtraction, including EO and EC conditions, were observed (Supplementary Table 1).

#### Motor single tasks (ST)

We did not find any significant effects on walking speed or sway distance under single walking (χ^2^ (4) = 1.51, p = 0.82, R^2^m = 0.24, R^2^c = 0.78), EO-standing (χ^2^ (4) = 4.05, p = 0.39, R^2^m = 0.03, R^2^c = 0.97), and EC-standing tasks (χ^2^ (4) = 1.58, p = 0.83, R^2^m = 0.07, R^2^c = 0.92). No significant alterations of muscle activities were found either (Supplementary Table 1).

#### Cognitive ST

**Fig. 7:**
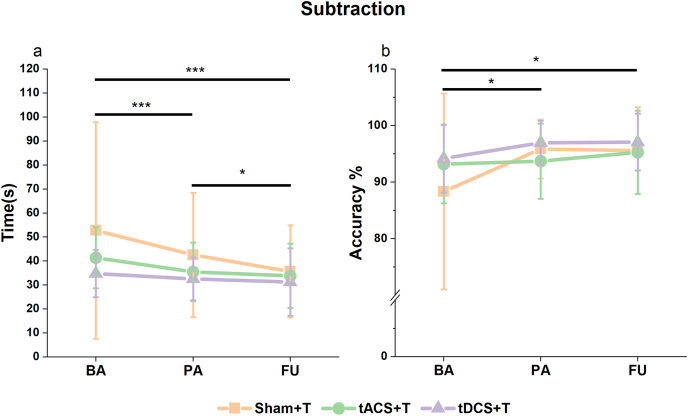
Subtraction performance under the ST condition. Lines represent different groups. Error bar represents mean ± standard deviation. **a**: Time to complete the subtraction task. **b**: Accuracy of the subtraction task. ∗: 0.01< p ≤ 0.05; ∗∗∗: p ≤ 0.001.

Intervention effect was observed via the discovery of the main Time effect on the subtraction task under the ST condition (χ^2^ (2) = 23.23, p < 0.001, R^2^m = 0.74, R^2^c = 0.95, Fig. 7a). Calculation speeds were significantly increased from BA to PA (estimate = −0.11, SE = 0.03, p < 0.001), BA to FU (estimate = −0.20, SE = 0.03, p < 0.001), and PA to FU (estimate = −0.08, SE = 0.03, p = 0.016) without any group differences. Moreover, results demonstrated improved accuracy from BA to PA (Time: χ^2^ (2) = 11.37, p = 0.003, R^2^m = 0.47, R^2^c = 0.51, estimate = 0.04, SE = 0.017, p = 0.046, Fig. 7b), and maintained until FU (estimate = 0.04, SE = 0.017, p = 0.025), no group differences were found though (Supplementary Table 1).

#### Self-report questionnaires

We did not find any significant interactions in fear of falling (F (4, 109.13) = 1.27, p = 0.28) and overall quality of life (χ^2^ (4) = 2.60, p = 0.62, R^2^m = 0.10, R^2^c = 0.78) (Supplementary Table 2).

#### Side effects/adverse events and blinding success

Only one participant in the combined sham stimulation group reported dizziness after two intervention sessions and subsequently withdrew from the study. No participant required any medical intervention following the stimulation. The most common side effect after stimulation was tingling, which was reported by 33.3% of participants in the Sham + training group, 42.1% of participants in the tACS group, and 27.7% in the tDCS group. Other adverse events were dizziness (11.1%, 5.3%, and 11.1% respectively), nervousness (11.1%, 10.5%, and 27.7% respectively), headache (11.1%, 15.8%, and 11.1% respectively), and fatigue (16.7%, 15.8%, and 16.7% respectively). We did not observe any skin redness after stimulation in any of the participants. The observed rate of correct guesses (34.5%, 19 out of 55, Sham + T: n = 6, tACS + T: n = 5, tDCS + T: n = 8) was significantly lower than the 50% chance level (χ^2^ = 5.25, p = 0.02), indicating that participants were not able to correctly identify whether they had received active or sham stimulation beyond random guessing.

## Discussion

This randomized, double-blind, sham-controlled study is the first to examine the effects of multi-session combining L-DLPFC tACS and task training on DT ability in healthy older adults. Participants completed 10 20min sessions and measurements were conducted before, after, and four weeks post-intervention using DT paradigms, single motor tasks, single cognitive tasks, and self-report questionnaires. Contrary to initial hypotheses, combining task training with either anodal tDCS or 4 Hz tACS did not show any superior benefits on DT compared to sham. All three groups showed cognitive improvements under ST and DT conditions, with no changes in motor tasks or significant group effects. Moreover, the combined intervention neither affected fear of falling nor health-related quality of life in participants.

We did not find any add-on effects of a 10-session, 20min L-DLPFC 4-Hz tACS or anodal tDCS to task training on DT performance in healthy elderly individuals. Indeed, similar results were reported in several previous studies [33,41]. For example, Ramasawmy et al. [42] employed concurrent anodal M1 tDCS and meditation in patients with fibromyalgia, significant lower pain sensation and higher life quality score were reported in both sham and real group after 10 intervention sessions. However, even though significant larger intracortical facilitation was identified in the real-combined group in this study, no statistical difference was identified in clinical outcomes between groups. Another study that utilized 10-session concurrent L-DLPFC anodal tDCS with cognitive training in patients with schizophrenia, results revealed significantly improved working memory performance in both real and sham groups, without any statistically significant group differences [43]. The add-on effect of theta-gamma tACS combined with cognitive training was explored in the healthy elderly, results suggested that episodic memory was improved in both sham and real conditions without group difference after 16 intervention sessions [44]. In addition, ceiling effects may have contributed to the absence of detectable group differences. Specifically, no significant changes were observed across the three groups in ST walking or ST EO standing task. At baseline, ST walking times were 18.03 s, 18.53 s, and 18.73 s in the three groups, and changed only marginally to 18.77 s, 19.96 s, and 18.76 s after the intervention. Similarly, ST EO-standing sway distances were 1047.65 mm, 1061.21 mm, and 1170.89 mm at baseline, and showed only minor changes to 1039.87 mm, 1058.58 mm, and 1266.95 mm after the intervention. Those limited changes suggested the performance on these motor tasks may have already reached a functional ceiling at baseline and cannot be further improvement through intervention in healthy older adults. On the other hand, it may also suggest that the motor component tasks employed in this study may not have been sufficiently demanding to induce measurable performance changes, limiting the sensitivity of these tasks to detect behavioral effects of the intervention. Previous studies have argued that simple psychomotor task, e.g., walking on a treadmill or on the flat ground does not threat the postural ability, may not have been sufficiently challenging to elicit age-related deficits specifically attributable to DT coordination [45,46].

Another possible explanation could be the ‘leaky-membrane effect’ [47,48], which has been frequently reported when tES is applied concurrently with ongoing motor activity. A growing body of evidence suggests that the physiological and behavioral effects of tES are strongly state-dependent [49,50], such that the same stimulation protocol may exert qualitatively different effects depending on the baseline level of neural activation. Specifically, previous studies have shown that anodal tDCS [51,52] or 140 Hz tACS [53] applied over the M1 increases cortical excitability in resting-state individuals assessed by transcranial magnetic stimulation induced motor-evoked potentials. In contrast, when stimulation is delivered during concurrent motor activity (e.g., finger movements), cortical excitability is often unchanged or even reduced. This interaction has been interpreted within a neurophysiological framework in which synaptic plasticity mechanisms, including long-term potentiation and depression, critically depend on intracellular Ca^2+^ concentrations [54,55]. Previous study addressed that during active motor engagement, intracellular Ca^2+^ levels may already be elevated, potentially driving the system closer to a saturation point. Under such conditions, additional field-induced depolarization may have limited capacity to further enhance excitability, or may even shift plasticity toward inhibitory processes [53]. Consistent with this interpretation, Moliadze et al. [56] demonstrated that 10 min of 140 Hz tACS at 1 mA over the left M1 robustly increased cortical excitability at rest, whereas this facilitation was markedly attenuated when stimulation was applied concurrently with a serial reaction time task. Taken together, these findings suggest that concurrent task engagement may constrain the modulatory range of tES, thereby reducing the likelihood of observing additive behavioral benefits when stimulation is combined with motor or cognitively demanding training.

Several studies have explored the effects of combined interventions on DT, including a single session of combined anodal tDCS of L-DLPFC and treadmill training in healthy older adults [30], a 36-session protocol combining 20min anodal L-DLPFC tDCS and TaiChi training in individuals with mild cognitive impairment [32], and a 12-session protocol comprising 20min anodal L-DLPFC tDCS followed by 30min treadmill training in older patients with PD [33]. Subtraction and walking tasks were applied concurrently. Results indicated an improvement in gait speed under DT conditions, with no evidence of placebo effects or cognitive enhancement. In contrast, our study revealed an opposite pattern: cognitive performance under DT conditions significantly improved following the intervention, whereas no change was observed in walking or standing performance. This inconsistency can be mainly related to the recruited populations. In our study, healthy older adults were recruited, while previous studies have demonstrated that task training induced greater optimization of motor function in geriatric patients with PD [57,58], osteoarthritis [59], mild cognition impairment [60], stroke [61], osteoporosis [62], etc. [17]. Evidence from a recent meta-analysis suggested that anodal tDCS targeting L-DLPFC enhanced DT gait performance in PD patients when compared with sham [63]. Another meta-analysis synthesized evidence from 88 randomized clinical trials (5522 participants) examining the effects of different forms of tES in patients with depressive disorders [64]. The analysis found that tDCS and tACS were associated with positive clinical outcomes and were generally well tolerated, with larger effect sizes observed when tDCS was combined with medication. Taken together, these observations indicate that combined intervention approaches may preferentially induce measurable benefits in populations with compromised neural resources, whereas effects in healthy older adults may be domain-specific. Other factors, including intervention sessions, training programs, and intervention durations, should also be taken into consideration when discussing the inconsistencies between the results of our work and those of previous works. For instance, the non-linear correlation between intervention session and performance was addressed before [65,66]. Hence, more studies should be conducted to better understand the role of different factors.

It was previously shown that the performances on STs such as walking, standing, or subtraction could not be modified through L-DLPFC tES, even though the same component task was improved under DT in healthy older adults [17]. Both single- [24,[67], [68], [69], [70]] and multi-session [71] studies reported similar findings, suggesting that the enhancement in dual-task performance was driven by an optimized recruitment of neural networks, rather than by improvements in the task process itself [22,70]. However, our study did not demonstrate any significant improvement in motor tasks, either ST or DT. A significant improvement in the subtraction task was observed from pre-to post-intervention across all groups, reflected by faster calculation speed and higher accuracy. Moreover, after the intervention, subtraction performance under walking and EO/EC standing DT conditions remained comparable to, or even slightly better than, that under the ST condition. These results indicate that multi-session interventions not only produce substantial improvements in behavioral performance but also promote adaptive changes in neural processes within DT condition [72,73].

Our study additionally identified a delayed effect of tDCS concurrently combined with training. More specifically, under EO-standing-subtraction and walking-subtraction conditions, improved cognitive performance was observed after the intervention and sustained through follow-up in both the tACS + T and sham + T groups, while improvements in the tDCS + T group emerged only at follow-up. This postponed effect aligns with a previous study [74]. In a study combining 10 sessions of 2 mA, 30min daily tDCS targeting the bilateral DLPFC with 4 × 60min sessions of cognitive behavioral therapy (twice/week) in patients with depression, the score of the Beck Depression Inventory was significantly lower at 12-week follow-up when compared to the sham group [75]. Neurophysiological evidence has suggested delayed excitability when stimulation occurs concurrently with motor activity [53]. However, we did not find a similar postponed effect in the EC-standing-subtraction condition, where all groups showed improvements in calculation speed after intervention. This may suggest that the delay effect induced by tDCS appears more likely under highly cognitively demanding conditions.

Another noteworthy finding is the inconsistency between the DTCs and the behavioral performances. Our study showed group-independent reductions in DTCs for motor tasks, but not for the cognitive task. Nevertheless, data analyses revealed no significant pre-to post-intervention changes in walking and standing performance during dual-task conditions, while cognitive performance improved significantly. It is important to note that the DTC formula reflects the relative percentage change of a given task from ST to DT conditions. Therefore, comparing DTCs only between pre- and post-intervention essentially examines the dynamic changes in this relative cost, rather than absolute performance differences. Some studies reported it as the primary outcome without addressing the performances of component tasks [[76], [77], [78]]. This may lead to misinterpretations, particularly when improvements are observed in both ST and DT performances, as the DTC then becomes less effective at detecting meaningful differences. This dissociation highlights the limitation of using DTC as a sole indicator of DT capacity. In our study, faster calculation speed was demonstrated in both DT and ST, followed by increased or maintained accuracy, but no alteration of DTCs was found. Hence, in order to better assess the DT ability, the performances of both component tasks should be reported, in case the improvement in one task was caused by the deterioration of the other.

Our findings must be interpreted in the light of the study's limitations. The current study did not include a control group that received only tES without any training. Therefore, it is not possible to definitively determine the specific effects of tES alone. We did not assess changes in cortical excitability after intervention, consequently missing the further investigation on the relationship between DT performance and neuroplastic changes. Future studies could incorporate transcranial magnetic stimulation to evaluate motor cortical excitability during DT performance. It should also be noted that the sham-combined group exhibited slightly poorer baseline performance on the primary outcome (walking–subtraction), suggesting a greater potential for improvement in this group. Although baseline performance was included as a covariate in the analyses to statistically account for pre-intervention differences across groups. Nevertheless, residual influences of baseline performance cannot be entirely excluded and should be considered when interpreting the magnitude of intervention effects. Moreover, the motor component tasks in our study may have been insufficiently challenging for healthy older adults, potentially leading to ceiling effects that masked further improvements induced by the intervention, therefore, more motor-demanding or cognitive-demanding motor tasks can be employed in future studies, such as walking on obstacle-laden tracks [45,46]. Integrating electroencephalography and functional magnetic resonance imaging would allow for a more comprehensive investigation of brain network dynamics and cognitive resource allocation during DT performance by combining event-related time-frequency analysis with high spatial precision [79]. Finally, future research should also consider variations in participant characteristics, electrode montages, and intervention durations to enhance generalizability and refine the understanding of tES-training interactions.

## Author contributions

Yong Jiang: Conceptualization, Investigation, Data analysis, Visualization, Writing – original draft.

Azade Lesani: Investigation, Writing – review & editing.

Xue Guo: Investigation, Writing – review & editing.

Isabella Wistuba: Investigation.

Raquel Guiomar: Data analysis, Writing – review & editing.

Denis Glage: Investigation, Writing – review & editing.

Perianen Ramasawmy: Conceptualization, Investigation, Writing – review & editing, Supervision.

Andrea Antal: Conceptualization, Writing – review & editing, Supervision, Project Administration.

## Sources of funding

No funding was provided for this study.

## Declaration of competing interest

The authors declare the following financial interests/personal relationships which may be considered as potential competing interests: P. Ramasawmy reports a relationship with Sooma Medical that includes: non-financial support. P. Ramasawmy reports a relationship with NeuroCare that includes: non-financial support. P. Ramasawmy reports a relationship with NeuroCare that includes: speaking and lecture fees. P. Ramasawmy reports a relationship with Quantalx Neuroscience Ltd that includes: non-financial support. A. Antal reports a relationship with European Society for Brain Stimulation that includes: board membership. A. Antal reports a relationship with The International Federation of Clinical Neurophysiology that includes: board membership. A. Antal reports a relationship with NeuroConn that includes: consulting or advisory. A. Antal reports a relationship with Electromedical Products International (Pulvinar) that includes: consulting or advisory. A. Antal reports a relationship with PlatoScience that includes: board membership. A. Antal reports a relationship with Sooma Medical that includes: non-financial support. If there are other authors, they declare that they have no known competing financial interests or personal relationships that could have appeared to influence the work reported in this paper.
