# Supplementary material for: Combining transcranial electrical stimulation with training in older adults: Effects on dual-task ability
Source: Neurotherapeutics. 2026 Mar 20;23(2):e00889. doi: 10.1016/j.neurot.2026.e00889 (PMC13069425; doi:10.1016/j.neurot.2026.e00889)
Supplement: Multimedia component 1 [file mmc1.docx]

Supplementary Table 1. Interaction and main effects of the secondary outcomes.

| **Parameters** | **Baseline control ^a^** | **Sham+T** | | | **tACS+T** | | | **tDCS+T** | | | **Interaction** | **Group effect** | **Time effect** | **R²m** | **R²c** |
| --- | --- | --- | --- | --- | --- | --- | --- | --- | --- | --- | --- | --- | --- | --- | --- |
|  |  | BA | PA | FU | BA | PA | FU | BA | PA | FU |  |  |  |  |  |
| STwalking (s) | N | 18.03 ± 1.90 | 18.77 ±2.60 | 18.17 ±2.30 | 19.53 ± 3.03 | 19.96 ± 3.73 | 19.59 ± 3.28 | 18.73 ±3.33 | 18.76 ±2.93 | 18.70 ±2.34 | χ² (4) = 1.51, p = 0.82 | χ² (2) = 2.59, p = 0.27 | χ² (2) = 3.24, p = 0.19 | 0.24 | 0.78 |
| STsubT (s) | Y | 52.69 ± 45.16 | 42.50 ± 25.93 | 35.58 ± 19.19 | 41.31 ±12.74 | 35.46 ± 12.22 | 33.76 ± 13.43 | 34.72 ± 9.84 | 32.51 ± 8.95 | 31.17 ± 14.06 | χ² (4) = 4.20, p = 0.37 | χ² (2) = 2.85, p = 0.24 | **χ² (2) = 23.23, p < 0.001** | 0.74 | 0.86 |
| STsubACC | N | 0.88 ± 0.17 | 0.95 ± 0.05 | 0.95 ± 0.07 | 0.93 ± 0.07 | 0.94± 0.06 | 0.95 ± 0.07 | 0.94 ± 0.06 | 0.97 ± 0.03 | 0.97 ± 0.05 | χ² (4) = 4.79, p = 0.30 | χ² (2) = 5.45, p = 0.06 | **χ² (2) = 11.37, p = 0.03** | 0.15 | 0.30 |
| ST EO-standing (mm) | N | 1047.65± 441.94 | 1039.87± 398.16 | 1112.65± 346.26 | 1061.21± 389.83 | 1058.58± 393.62 | 1122.95 ±351.91 | 1170.89± 373.14 | 1266.95± 396.05 | 1259.14± 302.99 | χ² (4) = 4.05, p = 0.39 | χ² (2) = 1.42, p = 0.48 | χ² (2) = 0.52, p = 0.76 | 0.03 | 0.97 |
| Tibialis anterior (uv) | Y | 12.76 ± 9.35 | 9.13 ± 4.08 | 7.72 ± 3.38 | 10.68 ± 18.30 | 8.95 ± 8.83 | 10.34 ± 8.44 | 8.08 ± 3.70 | 8.18 ± 2.55 | 8.62 ± 7.14 | χ² (4) = 6.77, p = 0.14 | **χ² (2) = 6.05, p = 0.05^c^** | χ² (2) = 3.48, p = 0.17 | 0.44 | 0.45 |
| Soleus (uv) | N | 14.10 ± 10.33 | 17.49 ± 9.19 | 18.00 ± 14.73 | 11.37 ± 9.52 | 13.25 ± 7.20 | 13.47 ± 6.72 | 15.61 ± 8.96 | 18.11 ± 9.56 | 13.98 ± 7.62 | χ² (4) = 3.06, p = 0.54 | χ² (2) = 2.72, p = 0.25 | χ² (2) = 4.00, p = 0.13 | 0.17 | 0.48 |
| ST EC-standing (mm) | N | 1281.58±502.20 | 1376.83±491.81 | 1377.26± 490.39 | 1318.41± 413.32 | 1358.09± 279.84 | 1294.87± 368.21 | 1370.44±386.03 | 1462.23± 415.04 | 1443.14± 298.03 | χ² (4) = 1.58, p = 0.83 | χ² (2) = 0.74, p = 0.69 | χ² (2) = 5.02, p = 0.08 | 0.07 | 0.92 |
| Tibialis anterior (uv) | N | 14.24 ± 10.56 | 16.87 ± 17.12 | 12.03 ± 7.60 | 16.54 ± 34.86 | 15.53 ± 26.16 | 18.58 ± 27.96 | 10.78 ± 7.23 | 10.30 ± 4.33 | 9.68 ± 5.94 | χ² (4) = 3.41, p = 0.49 | χ² (2) = 2.83, p = 0.24 | χ² (2) = 1.08, p = 0.58 | 0.11 | 0.55 |
| Soleus (uv) | N | 16.19 ± 12.93 | 18.29 ± 9.43 | 19.05 ± 16.49 | 12.25 ± 9.68 | 13.24 ± 6.88 | 16.10 ±10.73 | 15.96 ± 7.66 | 17.43 ± 8.70 | 14.10 ± 7.35 | χ² (4) = 4.60, p = 0.33 | χ² (2) = 3.23, p = 0.19 | χ² (2) = 1.47, p = 0.47 | 0.13 | 0.45 |
| DTCwalking | Y | 0.49 ± 0.34 | 0.32 ± 0.28 | 0.33 ± 0.21 | 0.31 ± 0.27 | 0.30 ± 0.37 | 0.30 ± 0.33 | 0.27 ± 0.31 | 0.20 ± 0.20 | 0.21 ± 0.18 | χ² (4) = 5.08, p = 0.27 | χ² (2) = 1.00, p = 0.60 | **χ² (2) = 10.49, p = 0.005** | 0.58 | 0.63 |
| DTC EO-standing | N | 0.44 ± 0.58 | 0.32 ± 0.31 | 0.29 ± 0.36 | 0.32 ± 0.51 | 0.32 ± 0.39 | 0.23 ± 0.29 | 0.31 ± 0.42 | 0.22 ± 0.21 | 0.14 ± 0.12 | χ² (4) = 3.38, p = 0.49 | χ² (2) = 1.28, p = 0.52 | **χ² (2) = 9.56, p = 0.008** | 0.04 | 0.85 |
| DTC EC-standing | N | 0.20 ± 0.26` | 0.08 ± 0.24 | 0.11 ± 0.31 | 0.14 ± 0.28 | 0.12 ± 0.22 | 0.14 ± 0.19 | 0.18 ± 0.26 | 0.09 ± 0.12 | 0.12 ± 0.16 | χ² (4) = 2.25, p = 0.68 | χ² (2) =0.01, p = 0.99 | **χ² (2) = 6.78, p = 0.03** | 0.04 | 0.60 |
| DTC subtraction | N | -0.09 ± 0.24 | -0.12 ± 0.21 | -0.005 ± 0.24 | -0.10 ± 0.17 | -0.11 ± 0.18 | -0.05 ± 0.18 | -0.02 ± 0.19 | -0.004 ± 0.28 | -0.07 ± 0.17 | χ² (4) = 4.05, p = 0.39 | χ² (2) = 2.41, p = 0.29 | χ² (2) = 2.84, p = 0.24 | 0.13 | 0.18 **^b^** |
| DTC EO-subtraction | N | -0.02 ± 0.23 | -0.10 ± 0.19 | -0.06 ± 0.14 | -0.08 ± 0.14 | -0.05 ± 0.11 | -0.06 ± 0.20 | -0.06 ± 0.10 | 0.02 ± 0.31 | -0.08 ± 0.15 | χ² (4) = 6.06, p = 0.19 | χ² (2) = 1.34, p = 0.51 | χ² (2) = 2.05, p = 0.36 | 0.09 | 0.22 |
| DTC EC- subtraction | N | -0.11 ± 0.24 | -0.09 ± 0.25 | -0.03 ± 0.04 | -0.10 ± 0.20 | -0.12 ± 0.15 | -0.11 ± 0.16 | -0.04 ± 0.15 | -0.04 ± 0.12 | -0.04 ± 0.13 | F (4,109.33) = 0.38,  p = 0.82 | F (2, 54.78) = 2.36,  p = 0.10 | F (2,109.34) = 0.43,  p = 0.65 | - | - |

a: baseline data as co-various or not; b: Non-significant in post-hoc; c: the random slope was dismissed for better modeling; STwalking: the time to complete 20m-walking with natural speed; STsubT: the time to complete subtraction task while sitting; STsubACC: the accuracy of subtraction task in ST condition; ST EO-standing: displacement of the center of pressure under single EO-standing; ST EC-standing: displacement of the center of pressure under single EC-standing; DTCwalking: DTC to walking from single walking to walking-subtraction condition; DTC EO-standing: DTC to standing task from single EO-standing to EO-standing-subtraction condition; DTC EC-standing: DTC to standing task from single EC-standing to EC-standing-subtraction condition; DTC subtraction: DTC to subtraction from single subtraction to walking-subtraction condition; DTC EO-subtraction: DTC to subtraction from single subtraction to EO-standing-subtraction condition; DTC EC-subtraction: DTC to subtraction from single subtraction to EC-standing-subtraction condition.
