# Supplementary material for: Combining transcranial electrical stimulation with training in older adults: Effects on dual-task ability
Source: Neurotherapeutics. 2026 Mar 20;23(2):e00889. doi: 10.1016/j.neurot.2026.e00889 (PMC13069425; doi:10.1016/j.neurot.2026.e00889)
Supplement: Multimedia component 2 [file mmc2.docx]

Supplementary Table 2. Analysis of self-report questionnaires.

| **Parameters** | **Baseline control^a^** | **Sham+T** | | | **tACS+T** | | | **tDCS+T** | | | **Interaction** | **Group** | **Time** | **R²m** | **R²c** |
| --- | --- | --- | --- | --- | --- | --- | --- | --- | --- | --- | --- | --- | --- | --- | --- |
|  |  | BA | PA | FU | BA | PA | FU | BA | PA | FU |  |  |  |  |  |
| **FESI** | N | 17.77 ± 2.18 | 17.83 ± 2.06 | 17.22 ±2.04 | 18.42 ± 3.65 | 19.15 ± 4.40 | 19.36 ± 4.52 | 18.50 ± 4.17 | 18.66 ± 3.28 | 18.11 ± 2.89 | F (4, 109.13) = 1.27,  p = 0.28 | F (2, 55.04) = 0.78  p = 0.46 | F (2, 109.13) = 0.71,  p = 0.49 | - | - |
| **SF36** | N | 82.20 ± 8.46 | 82.62 ± 9.36 | 84.76 ± 7.07 | 76.41 ± 12.84 | 76.02 ± 14.38 | 75.41 ± 19.26 | 75.56 ± 14.01 | 73.29 ± 13.17 | 74.68 ± 16.07 | χ² (4) = 2.60, p = 0.62 | χ² (2) = 2.29, p = 0.31 | χ² (2) = 1.17, p = 0.55 | 0.10 | 0.78 |
| SFPF | N | 93.33 ± 8.57 | 92.50 ± 7.52 | 94.72 ± 7.16 | 87.89 ± 12.39 | 88.02 ± 14.30 | 86.57 ± 12.91 | 85.83 ± 19.87 | 84.81 ± 16.90 | 85.71 ± 16.82 | χ² (4) = 1.38, p = 0.84 | χ² (2) = 3.21, p = 0.20 | χ² (2) = 0.78, p = 0.67 | 0.09 | 0.82 |
| SFPH | N | 92.36 ± 11.45 | 93.05 ± 14.36 | 97.22 ± 8.08 | 79.47 ± 30.13 | 89.47 ± 28.03 | 75.00 ± 39.08 | 79.16 ± 32.36 | 77.77 ± 35.23 | 75.00 ± 34.23 | χ² (4) = 4.67, p = 0.32 | χ² (2) =2.37, p = 0.30 | χ² (2) = 0.61, p = 0.73 | 0.11 | 0.50 |
| SFE | N | 94.44 ± 17.15 | 89.83 ± 19.06 | 100 ± 0 | 87.71 ± 22.81 | 80.70 ± 33.91 | 82.45 ± 35.77 | 85.18 ± 32.78 | 70.92 ± 34.86 | 80.41 ± 33.44 | χ² (4) = 2.59, p = 0.62 | χ² (2) =4.77, p = 0.09 | χ² (2) = 5.92, p = 0.51 | 0.08 | 0.50 |
| SFF | N | 74.44 ± 10.55 | 78.33 ± 13.39 | 74.94 ± 14.16 | 68.94 ± 17.28 | 68.42 ± 19.44 | 70.00 ± 20.88 | 65.00 ± 10.71 | 65.55 ± 13.81 | 65.44 ± 15.00 | χ² (4) = 2.06, p = 0.72 | χ² (2) =2.92, p = 0.23 | χ² (2) = 1.57, p = 0.45 | 0.07 | 0.78 |
| SFWB | N | 66.44 ± 21.30 | 69.55 ± 20.07 | 67.55 ± 18.40 | 65.47 ± 19.92 | 61.05 ± 20.12 | 63.78 ± 21.88 | 67.11 ± 16.72 | 67.11 ± 17.11 | 69.41 ± 18.59 | F (4, 108.99) = 2.06,  p = 0.99 | F (2, 54.96) = 0.34,  p = 0.71 | F (2, 108.99) = 0.11,  p = 0.89 | - | - |
| SFSF | N | 94.44 ± 10.69 | 91.94 ± 13.76 | 97.91 ± 4.79 | 88.15 ± 15.85 | 87.50 ± 16.66 | 90.13 ± 12.20 | 85.41 ± 16.74 | 81.52 ± 16.45 | 80.58 ± 19.15 | χ² (4) = 3.27, p = 0.51 | χ² (2) =3.18, p = 0.20 | χ² (2) = 2.20, p = 0.33 | 0.13 | 0.47 |
| SFP | N | 82.08 ± 14.75 | 84.30 ± 15.54 | 85.97 ± 16.63 | 79.21 ± 19.04 | 78.68 ± 20.07 | 79.34 ± 19.57 | 79.30 ± 14.67 | 77.22 ± 15.83 | 79.41 ± 15.37 | χ² (4) = 0.53, p = 0.97 | χ² (2) = 0.53, p = 0.76 | χ² (2) = 0.46, p = 0.79 | 0.04 | 0.46 |
| SFGH | N | 60.00 ± 19.25 | 61.38 ± 22.41 | 59.72 ± 21.92 | 54.47 ± 21.91 | 54.21 ± 22.37 | 56.05 ± 24.01 | 57.50 ± 15.07 | 61.38 ± 14.01 | 61.47 ± 17.11 | F (4, 109.04) = 0.68,  p = 0.60 | F (2, 55.00) = 0.51,  p = 0.60 | F (2, 109.04) = 0.95,  p = 0.39 | - | - |

a: baseline data as co-various or not; FESI: falls efficiency scale questionnaire; SF36: Score of the short-form life quality questionnaire; SFPF: Physical functioning within SF36; SFPH: Role limitation due to physical health; SFE: Role limitation due to emotional problems; SFF: Fatigue; SFWE: Emotional well-being; SFSF: Social functioning; SFP: Pain; SFGH: General health.
