# Supplementary material for: Combining transcranial electrical stimulation with training in older adults: Effects on dual-task ability
Source: Neurotherapeutics. 2026 Mar 20;23(2):e00889. doi: 10.1016/j.neurot.2026.e00889 (PMC13069425; doi:10.1016/j.neurot.2026.e00889)
Supplement: Multimedia component 3 [file mmc3.docx]

**Supplementary material**

All mixed-effects analyses were conducted in R (version 4.5.1) and RStudio. Linear mixed-effects models were fitted using the lme4 (version 1.1-37) and lmerTest (version 3.1-3) packages. Generalized linear mixed-effects models were fitted using glmmTMB (version 1.1.11). Post-hoc comparisons were conducted using emmeans (version 1.11.2). Effect sizes and model diagnostics were obtained using effectsize (version 1.0.1), MuMIn (version 1.48.11), performance (version 0.15.0), and car (version 3.1-3).

Supplementary Table 3. Mixed-effects models used for statistical analysis.

| Parameter | ModelType | Family | Link | Formula |
| --- | --- | --- | --- | --- |
| STwalking | GLMM | Gamma | log | ~ Group * Visit + Age + Sex + MoCA + (1\|ID) |
| DTwalking | GLMM | Gamma | log | ~Group * Visit + Age + Sex + MoCA + (1\|ID) |
| STsubT | GLMM | Gamma | log | ~ Group * Visit + Age + Sex + MoCA + Baseline + (1\|ID) |
| STsubAcc | GLMM | Gamma | log | ~ Group * Visit + Age + Sex + MoCA + (1\|ID) |
| DTsubT | GLMM | Gamma | log | ~ Group * Visit + Age + Sex + MoCA + Baseline + (1\|ID) |
| DTsubAcc | GLMM | Gamma | log | ~ Group * Visit + Age + Sex + MoCA + (1\|ID) |
| DT EOsubT | GLMM | Gamma | log | ~ Group * Visit + Age + Sex + MoCA +Baseline + (1\|ID) |
| DT EOsubAcc | GLMM | Gamma | log | ~ Group * Visit + Age + Sex + MoCA + Baseline + (1\|ID) |
| DT ECsubT | GLMM | Gamma | log | ~ Group * Visit + Age + Sex + MoCA + Baseline + (1\|ID) |
| DT ECsubAcc | GLMM | Gamma | log | ~ Group * Visit + Age + Sex + MoCA + (1\|ID) |
| ST EOstanding | GLMM | Gamma | log | ~ Group * Visit + Age + Sex + MoCA + (1\|ID) |
| ST ECstanding | GLMM | Gamma | log | ~ Group * Visit + Age + Sex + MoCA + (1\|ID) |
| DT EOstanding | GLMM | Gamma | log | ~ Group * Visit + Age + Sex + MoCA + (1\|ID) |
| DT ECstanding | GLMM | Gamma | log | ~ Group * Visit + Age + Sex + MoCA + (1\|ID) |
| FESI | LMM | gaussian | identity | ~ Group * Visit + Age + Sex + MoCA + (1\|ID) |
| SFPF | GLMM | Gamma | log | ~ Group * Visit + Age + Sex + MoCA + (1\|ID) |
| SFPH | GLMM | Gamma | log | ~ Group * Visit + Age + Sex + MoCA + (1\|ID) |
| SFE | GLMM | Gamma | log | ~ Group * Visit + Age + Sex + MoCA + (1\|ID) |
| SFF | GLMM | Gamma | log | ~ Group * Visit + Age + Sex + MoCA + (1\|ID) |
| SFWB | LMM | gaussian | identity | ~ Group * Visit + Age + Sex + MoCA + (1\|ID) |
| SFSF | GLMM | Gamma | log | ~ Group * Visit + Age + Sex + MoCA + (1\|ID) |
| SFP | GLMM | Gamma | log | ~ Group * Visit + Age + Sex + MoCA + (1\|ID) |
| SFGH | LMM | gaussian | identity | ~ Group * Visit + Age + Sex + MoCA + (1\|ID) |
| DTCwalking | GLMM | Gamma | log | ~ Group * Visit + Age + Sex + MoCA + Baseline + (1\|ID) |
| DTCsub | GLMM | Gamma | log | ~ Group * Visit + Age + Sex + MoCA + (1\|ID) |
| DTC EOsub | GLMM | Gamma | log | ~ Group * Visit + Age + Sex + MoCA + (1\|ID) |
| DTC ECsub | LMM | gaussian | identity | ~ Group * Visit + Age + Sex + MoCA + (1\|ID) |
| DTC EOstanding | GLMM | Gamma | log | ~ Group * Visit + Age + Sex + MoCA + (1\|ID) |
| DTC ECstanding | GLMM | Gamma | log | ~ Group * Visit + Age + Sex + MoCA + (1\|ID) |
| ST EOTA | GLMM | Gamma | log | ~ Group * Visit + Age + Sex + MoCA + Baseline |
| ST EOSO | GLMM | Gamma | log | ~ Group * Visit + Age + Sex + MoCA + (1\|ID) |
| ST ECTA | GLMM | Gamma | log | ~ Group * Visit + Age + Sex + MoCA + (1\|ID) |
| ST ECSO | GLMM | Gamma | log | ~ Group * Visit + Age + Sex + MoCA + (1\|ID) |
| DTEOTA | GLMM | Gamma | log | ~ Group * Visit + Age + Sex + MoCA + (1\|ID) |
| DTEOSO | GLMM | Gamma | log | ~ Group * Visit + Age + Sex + MoCA + Baseline + (1\|ID) |
| DTECTA | GLMM | Gamma | log | ~ Group * Visit + Age + Sex + MoCA + (1\|ID) |
| DTECSO | GLMM | Gamma | log | ~ Group * Visit + Age + Sex + MoCA + (1\|ID) |
| SF36 | GLMM | Gamma | log | ~ Group * Visit + Age + Sex + MoCA + (1\|ID) |

Baseline values were included as covariates for parameters showing either marginal or significant group differences at baseline.
